# First description of the female of the jumping spider *Balmaceda nigrosecta* Mello-Leitão (Salticidae, Dendryphantini, Marpissina)

**DOI:** 10.3897/zookeys.563.6705

**Published:** 2016-02-15

**Authors:** Gonzalo D. Rubio, Julián E. Baigorria, G. B. Edwards

**Affiliations:** 1 National Research Council (CONICET); 2 Instituto de Biología Subtropical, Universidad Nacional de Misiones (IBS, UNaM), Puerto Iguazú, Misiones, Argentina; 3 Florida State Collection of Arthropods, Division of Plant Industry, Gainesville, Florida, USA

**Keywords:** Natural history, salticid, taxonomy

## Abstract

The female of *Balmaceda nigrosecta* Mello-Leitão, 1945 is described and illustrated for the first time. In addition, this paper further illustrates the male, and provides the first known observations on the natural history of this species, including habitat, cohabitation, and prey capturedata.

## Introduction

Jumping spiders (Salticidae) constitute a relatively young family, which has rapidly radiated (Bodner and Maddison 2012; Hill and Edwards 2013). This is the largest family of spiders, with 5838 described species (WSC 2015). However, one major problem is that this diversity may be hyper-inflated due to lack of matching of single-sex known species (Edwards 2014). This problem, in part, may have contributed to some quantitative inconsistencies between salticid databases, as observed among those of Prószyński (2015), the WSC (2015), and Metzner (2015).

In Prószyński’s database, accepted species status means that both sexes are adequately described and illustrated, while incomplete is due to the description and illustration of only one sex. A proposal for matching sexes was given by Edwards (2014) that consists in searching for an autapomorphy shared by both sexes as an intraspecific counterpart to an interspecific synapomorphy, considering also geographical and other data in an analysis (Edwards 2014). Use of this methodology is strengthened when the species in question belongs to a group that is already well-defined by morphology that agrees with its phylogenetic placement based on molecular data, e.g. Marpissinae (Maddison et al. 2014) [recently reclassified as Subtribe Marpissina (Maddison 2015)].

The marpissine genus *Balmaceda* Peckham & Peckham, 1894 is exclusive to the Americas. It currently includes eight valid species (WSC 2015; Metzner 2015), of which only the type species is known for both sexes, and five are known only for male or female (WSC 2015; the sixth is a nomen dubium). The latter is the case for *Balmaceda nigrosecta* Mello-Leitão, 1945. These spiders are similar to other marpissines as *Metacyrba* and *Platycryptus* (see Edwards 2006) in body and external genitalia forms. There is also some similarity to the euophryine *Corticattus* in the body form, but an examination of the genitalia can easily distinguish them (see Zhang and Maddison 2012).

In recent surveys of the salticid fauna from Misiones, in Northeastern Argentina (Rubio 2014), males and females of *Balmaceda nigrosecta* were observed and collected together. The coexistence of male and female in the same retreat observed in the field provided definitive evidence for conspecificity in our samples. In this paper, the female of *Balmaceda nigrosecta* is described for the first time and its somatic and genital morphology is illustrated. Some data on natural history are also presented.

## Methods

Field observations of living specimens were made in Misiones Province, Northeastern Argentina. Specimens were collected on walls of brick houses in Iguazú National Park and peri-urban habitats of Puerto Iguazú. This area corresponds to the Upper Parana Atlantic Forest Eco-region (Olson et al. 2001).

Morphological terms and description formats follow the main recent works about marpissines (Edwards 2006) and similar jumping spiders (Ruiz and Brescovit 2013). Female genitalia were cleared in clove oil to study the internal structures after digestion in a hot 10–20% KOH solution (Ramírez 2014). Temporary preparations were examined using a Leica DM500 compound microscope and a Leica M60 stereomicroscope. All measurements are in millimeters, and were obtained with an ocular micrometer following Ruiz and Brescovit (2013). Leg segments are measured for length, except the first two femora and tibiae which are measured length x width. Photographs in nature were taken with a Nikon D80 digital camera using a Micro-Nikkor 85 mm lens. Specimens examined are deposited at the arachnological collections of the Instituto de Biología Subtropical, Misiones (IBSI-Ara, G. Rubio).

Abbreviations used are updated, following Zhang and Maddison (2015):

AG
 accessory gland 


AT
 atrium 


CD
 copulatory duct 


CO
 copulatory opening 


E
 embolus 


FD
 fertilization duct 


RTA
 retrolateral tibial apophysis 


S
 spermatheca 


SP
 spermophore 


T
 tegulum 


## Results

### Taxonomy Family Salticidae Blackwall, 1841 Subfamily Salticinae Blackwall, 1841 Tribe Dendryphantini Menge, 1879 Subtribe Marpissina Simon, 1901 Genus *Balmaceda* Peckham & Peckham, 1894

#### Balmaceda nigrosecta

Taxon classificationAnimaliaAraneaeSalticidae

Mello-Leitão, 1945

Figs 1–5 , 6–11

Balmaceda nigrosecta Mello-Leitão, 1945: 277.
Metacyrba nigrosecta , Galiano 1980: 35.
Balmaceda nigrosecta , Edwards 2006: 211, figs 123–126; Rubio 2014: 7, fig 11; World Spider Catalog 2015.

##### Material examined.

Argentina: Misiones: 1 ♂ (holotype), Puerto Victoria, S26.33441°, W54.65540°, VI.1943, Zenzes leg. (MLP 16710; examined); 1 ♀, Iguazú National Park, Centro de Investigaciones Ecológicas Subtropicales, -25.67859°, -54.44927°, 20.IX.2014, G.D. Rubio leg. (IBSI-Ara 00198; tissue sample GDR 4126); 1 ♂ and 1 ♀, same locality, 17.X.2014, G.D. Rubio leg. (IBSI-Ara 00207); 2 ♀, same locality, 4.III.2015, G.D. Rubio leg. (IBSI-Ara 00291; tissue sample GDR 4143); 1 ♂, Puerto Iguazú, -25.59351°, -54.56968°, 20.XII.2014, J. Baigorria leg. (IBSI-Ara 00246).

##### Diagnosis.

**Figure 1–5.**
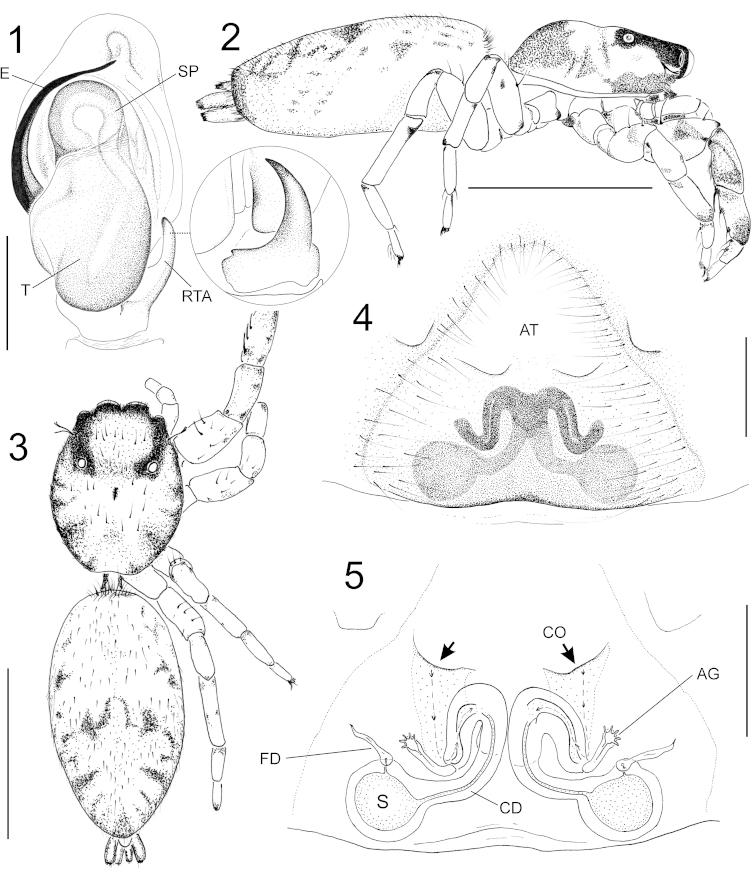
*Balmaceda nigrosecta* Mello-Leitão. **1** Male palp, ventral view (inset with close-up of RTA in retrolateral view) **2** female, lateral habitus **3** same, dorsal habitus **4** epigyne, ventral view **5** same, cleared. (AG–accessory gland; AT–atrium; CD–copulatory duct; CO–copulatory opening; E–embolus; FD–fertilization duct; RTA–retrolateral tibial apophysis; S–spermatheca; SP–spermophore; T–tegulum). Scale bars: 0.35 mm (**1**); 3 mm (**2**, **3**); 0.2 mm (**4**, **5**).

Specimens of *Balmaceda nigrosecta* resemble *Balmaceda picta* Peckham & Peckham, 1894 and *Balmaceda reducta* Chickering, 1946 in general body coloration (Chickering 1946; Edwards 2006: figs 115, 118, 123), and in general structure of epigyne, having an anterior atrium with narrow sclerotized rims of the copulatory openings on the posterior edge of the atrium (Figs 4, 5; arrow, rim of CO) (Chickering 1946: fig 47; Edwards 2006: fig 116), respectively. It can be distinguished from *Balmaceda picta* by having a larger atrium, with the copulatory openings (CO) farther apart, anteriorly concave, and nearly transverse in orientation (*Balmaceda picta* has the COs nearly touching, anteriorly convex, and strongly oblique in orientation), and a thinner and curved male retrolateral tibial apophysis (Figs 1, 4, 5; compare with Edwards 2006: figs 116, 121, 122, 126). Also, the “W” shaped transverse mark across the middle of the abdomen is distinctive for both sexes, as only the lateral parts of this mark are evident and the medial connecting parts are absent for *Balmaceda picta*. It can be distinguished from *Balmaceda reducta* by having the copulatory ducts (CD) contiguous along the mid-line of the body in the middle of the duct (Fig. 5; compare with Chickering 1946: fig 47).

##### Description.

**Figure 6–11.**
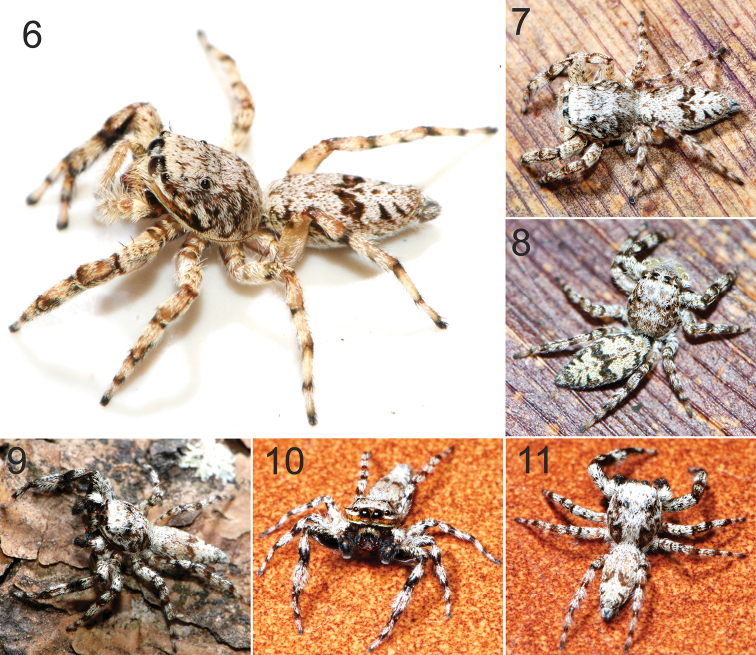
*Balmaceda nigrosecta* Mello-Leitão, habitus of living specimens from Iguazú National Park. Female (**6–8**); male (**9–11**).

Female from Iguazú National Park (IBSI-Ara 00207) (Figs 2–8). Total length: 7.84. Carapace length: 3.10; width: 2.44; height: 1.00. Carapace low, reddish brown, darker toward the borders and in the cephalic region, covered with white scales and sparse black hairs (Figs 6–8). Length of the dorsal eye field: 1.30. Width of the anterior eye row: 1.65; posterior: 1.45. Clypeus very low (0.05 height), with white hairs. Chelicera dark orange, vertical, with two teeth on promargin and one bicuspid tooth on retromargin. Labium and endites brown, sternum lighter. Palp yellow. Leg I stout, especially tibia and femur. Legs 4123, light brown with scattered dark spots usually where legs articulate, covered with sparse black hairs. Prolateral ventral margin of leg I spotted with brown, mainly on femur, patella and proximal tibia. Femur I 1.68×0.76; II 1.45x0.60; III 1.32; IV 1.67. Patella I 1.15; II 0.92; III 0.80; IV 0.97. Tibia I 1.25x0.42; II 1.00x0.35; III 0.83; IV 1.45. Metatarsus I 0.82; II 0.80; III 0.92; IV 1.09. Tarsus I 0.42; II 0.42; III 0.50; IV 0.52. Leg macrosetae: femur I, II d 1-1-p1, p 0-1-2(d1+v1); III d 1-1-p1, r 0-1-2(d1+v1); IV d 1-1-1. Tibia I v 2-2-2, p 0-1; II v 2-2-2; III, IV v p1di; Metatarsus I, II v2-2; III, IV v 2di, r 1di, p 1di. Abdomen length: 4.20; width: 2.32. Abdomen oval, with sparse black hairs; coloration pale yellow with a brown irregular W-shaped mark in middle of dorsum (Figs 6–8); margins spotted with brown, and four short bands inclined posterolaterally (Figs 6–8); venter pale yellow. Epigyne (Figs 4, 5): epigynal plate large, with a broad anterior atrium (a wide, shallow concavity); atrium shaped like a broad inverted V, each branch of which intersects (in a position just anterior to median) a slightly oblique slit-like copulatory opening that has a narrow strongly sclerotized rim (Fig. 5); copulatory ducts short, with a 180° bend backwards, connecting to a spherical spermatheca. An accessory gland (AG) occurs posterior to copulatory opening at posterior end of copulatory duct head (region of duct from CO to gland and first bend in duct). Fertilization duct anterior to spermatheca (Fig. 5). Spinnerets pale yellow. Variability: without significant variation in color pattern (n=4), otherwise total length: 6.19–7.84; carapace length: 2.65–3.10, width: 2.15–2.44, height: 0.87–1.12; abdomen length: 3.36–4.80, width: 1.70–2.70; epigynal plates may vary slightly in amount of sclerotization, and some abdomens are more pigmented than others.

Male (Holotype, MLP 16710): See Mello-Leitão (1945: 277) and Edwards (2006: 211, figs 123–126). Left palp as in figure 1, habitus as in figures 9–11.

##### Comments.

From the illustrations in the original description it is clear that this species is very closely related to *Balmaceda reducta* (Chickering 1946: 64), and *Balmaceda nigrosecta* possibly is a senior synonym of *Balmaceda reducta*. Because the known distribution of *Balmaceda reducta* is limited (Panama) and far from Argentina, no synonymy will be made, as the genus is not yet adequately sampled in the region.

Because we note that the morphological data are quite similar among members of the genus, it is difficult to establish an intersexual autapomorphy (see Edwards 2014) for *Balmaceda nigrosecta*. Nevertheless, it appears that the irregular W-shaped mark in the middle of the abdominal dorsum is a species shared autapomorphy(Figs 6–11). Sex matching is also supported by geographic and phenological evidence, and by an instance of both sexes co-habiting in the same retreat, where an adult male and an adult female were found together.

##### Natural history.

**Figure 12–17.**
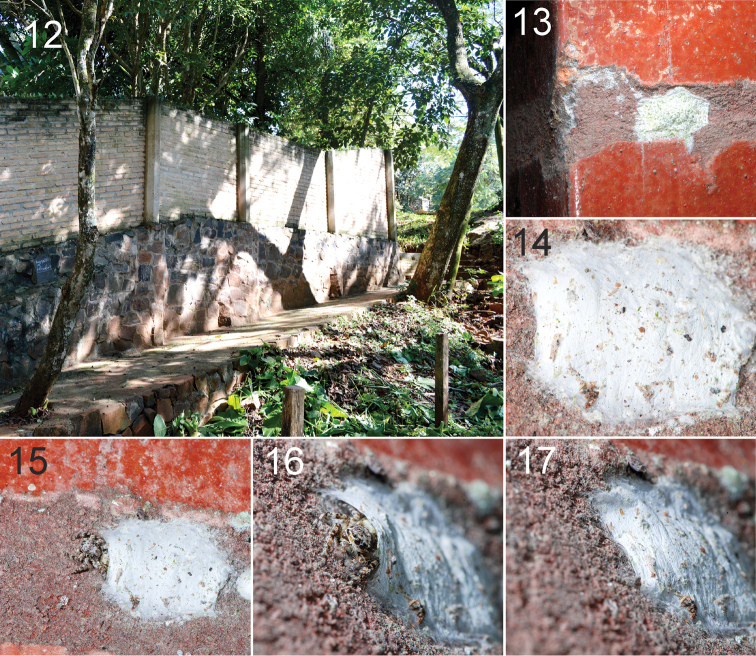
Habitat and natural retreat of *Balmaceda nigrosecta* Mello-Leitão. Puerto Iguazú (**12**), Iguazú National Park (**13–17**). Note the typical stalking position in **16**.

*Balmaceda nigrosecta* has sexual dimorphism as frequently occurs in other salticids, althoughstrong dimorphism is uncommon in marpissines. In this case the sexual dimorphism is weak; the males only show a slightly darker color in palps and first pair of legs. They live in many parts of the peri-urban area (Fig. 12), even on light poles. They make a flat retreat or nest, about 15 mm long, always placed perpendicular to the ground, between 1 and 2 meters above ground (Figs 13–17). The entrance opening can be on either side. Spiders are positioned at the entrance, with the carapace leaning out (Figs 15, 16), usually looking down (as figure 16). In fifty-one observations, we saw them hunt in the same way: locate prey from the retreat or while actively searching (sometimes we observed them walking in the vicinity of the retreat in search of prey); when they detected something moving, they would accelerate towards it. Here one of two things would happen: if it was an unpalatable prey (e.g. an ant), spiders did not attack and returned quickly to the retreat; but if it was a potential prey, the spiders accelerated a definite distance, about 10 body lengths, and then lowered themselves close to the substrate, and continued approaching very slowly, like a cat stalking, then jumped extremely quickly over the prey. The catch was always observed to be successful, in one movement.

##### Distribution.

Only known from northeast Argentina, in Misiones Province.

## Supplementary Material

XML Treatment for Balmaceda nigrosecta
